# Adeno-associated virus-mediated CASQ2 delivery rescues phenotypic alterations in a patient-specific model of recessive catecholaminergic polymorphic ventricular tachycardia

**DOI:** 10.1038/cddis.2016.304

**Published:** 2016-10-06

**Authors:** Francesco Lodola, Diego Morone, Marco Denegri, Rossana Bongianino, Hiroko Nakahama, Lucia Rutigliano, Rosanna Gosetti, Giulia Rizzo, Alessandra Vollero, Michelangelo Buonocore, Carlo Napolitano, Gianluigi Condorelli, Silvia G Priori, Elisa Di Pasquale

**Affiliations:** 1 Molecular Cardiology, IRCCS Fondazione Salvatore Maugeri, Pavia, Italy; 2 Laboratory of Inflammation and Immunology in Cardiovascular Pathologies, Humanitas Research Hospital, Rozzano (MI), Italy; 3 Institute of Genetic and Biomedical Research (IRGB) – UOS Milan, National Research Council of Italy, Milan, Italy; 4 Unit of Clinical Neurophysiology & Neurodiagnostic Skin Biopsy, IRCCS Fondazione Salvatore Maugeri, Pavia, Italy; 5 Humanitas University, Rozzano (MI), Italy; 6 Department of Molecular Medicine, University of Pavia, Pavia, Italy

## Abstract

Catecholaminergic Polymorphic Ventricular Tachycardia type 2 (CPVT2) is a highly lethal recessive arrhythmogenic disease caused by mutations in the calsequestrin-2 (*CASQ2*) gene. We have previously demonstrated that viral transfer of the wild-type (WT) *CASQ2* gene prevents the development of CPVT2 in a genetically induced mouse model of the disease homozygous carrier of the R33Q mutation. In the present study, we investigated the efficacy of the virally mediated gene therapy in cardiomyocytes (CMs) differentiated from induced pluripotent stem cells (iPSCs) obtained from a patient carrying the homozygous *CASQ2-G112+5X* mutation. To this end, we infected cells with an Adeno-Associated Viral vector serotype 9 (AAV9) encoding the human *CASQ2* gene (AAV9-h*CASQ2*). Administration of the human WT *CASQ2* gene was capable and sufficient to restore the physiological expression of calsequestrin-2 protein and to rescue functional defects of the patient-specific iPSC-derived CMs. Indeed, after viral gene transfer, we observed a remarkable decrease in the percentage of delayed afterdepolarizations (DADs) developed by the diseased CMs upon adrenergic stimulation, the calcium transient amplitude was re-established and the density and duration of calcium sparks were normalized. We therefore demonstrate the efficacy of the AAV9-mediated gene replacement therapy for CPVT2 in a human cardiac-specific model system, supporting the view that the gene-therapy tested is curative in models with different human mutations of CPVT.

Catecholaminergic Polymorphic Ventricular Tachycardia type 2 (CPVT2 – OMIM 611938) is a rare form of life-threating arrhythmia caused by mutations in the gene encoding calsequestrin-2 (*CASQ2)*, a luminal Ca^2+^ binding protein within the sarcoplasmic reticulum (SR).^1^ CASQ2 binds and sequesters Ca^2+^ within the SR, releasing it during the systolic phase of contraction. CPVT2 is inherited with an autosomal recessive pattern and is clinically characterized by episodic syncope and/or life-threatening cardiac arrhythmias induced by adrenergic activation, such as upon exercise or emotional stress. It usually occurs in children or young adults with no evidence of structural abnormalities of the heart, 30–35% of which undergo a fatal arrhythmic event before the age of 35. Approximately 25% of patients do not respond to antiadrenergic therapy and need implantable cardioverter-defibrillators (ICDs).^2, 3^ ICDs, though life-saving, carry the risk of adverse events such as infection, inappropriate shocks, and rupture of leads that may require ICD explant.

Gene therapy may thus represent an innovative therapeutic strategy for hereditary tachyarrhythmias.^4^

On this subject, our group has demonstrated that viral-mediated administration of the wild-type (WT) *CASQ2* gene was able to prevent the development of the disease in a *CASQ2* knockout mouse model and in mice knocked-in for the homozygous CASQ2-R33Q mutation.^5, 6, 7^ In this early study, however, we did not study whether the strategy was effective also in an experimental model based on human cells. Indeed, a critical step in devising a clinically usable gene therapy for CPVT is to demonstrate that the vector is effective in human cells and independently of the specific mutation. Thus, we decided to test whether AAV9-based CASQ2 delivery reverts the disease phenotype in the human setting, studying a *CASQ2* homozygous G112+5X nonsense mutation, we had previously found in a family suffering from CPVT2 and extensively characterized in rat cardiomyocytes (CMs).^8^

To this end, we employed induced Pluripotent Stem Cells (iPSCs), an increasingly used model system to study human inherited cardiovascular diseases, in particular primary cardiomyopathies and arrhythmogenic diseases, including Long QT and Brugada syndromes as well as CPVT.^9, 10, 11, 12, 13, 14, 15, 16, 17^ We have previously generated an iPSC-based model for the autosomal dominant form of the disease, CPVT1 (which is linked to mutation of the cardiac ryanodine receptor, *RyR2*), demonstrating that inhibition of Calcium–Calmodulin-dependent Kinase II (CAMKII) restored the normal electrophysiological phenotype.^4^

Here, we took advantage of the iPSC technology to generate a cardiac model of CPVT2 from a patient carrying the G112+5X nonsense mutation and tested the efficacy of AAV9-based CASQ2 delivery on cells phenotype. The results of the present study support the notion that AAV9-mediated gene therapy can be used on human cells to reinstate CASQ2 functionality.

## Results

### Generation of the human cardiac model of CPVT2

**Figure 1:**
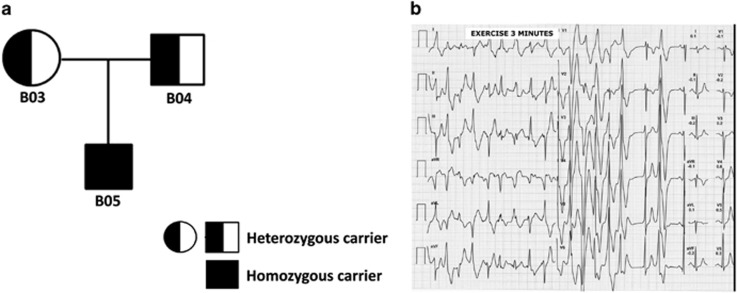
Pedigree and clinical phenotype of the CPVT family. (**a**) Pedigree of the recessive CPVT family investigated in this study. B05 is the proband, who is homozygous (HO) for the mutation and clinically affected, whereas heterozygous kin are not clinically affected; square=male; circle=female. (**b**) Bidirectional ventricular tachycardia recorded off therapy in the proband (paper speed, 25 mm/s)

To assess the efficacy of biological therapy for CPVT2, we developed a human model of the disease by applying iPSC technology to a family in which we identified a deletion of 16 nucleotides (c.339–354, p.G112+5X) in the *CASQ2* gene^8^ (Figure 1a). This mutation generates a premature stop codon and, hence, a lack of protein expression. The clinical counterpart is a life-threatening phenotype of bidirectional ventricular tachycardia in response to catecholaminergic stress (Figure 1b).

**Figure 2:**
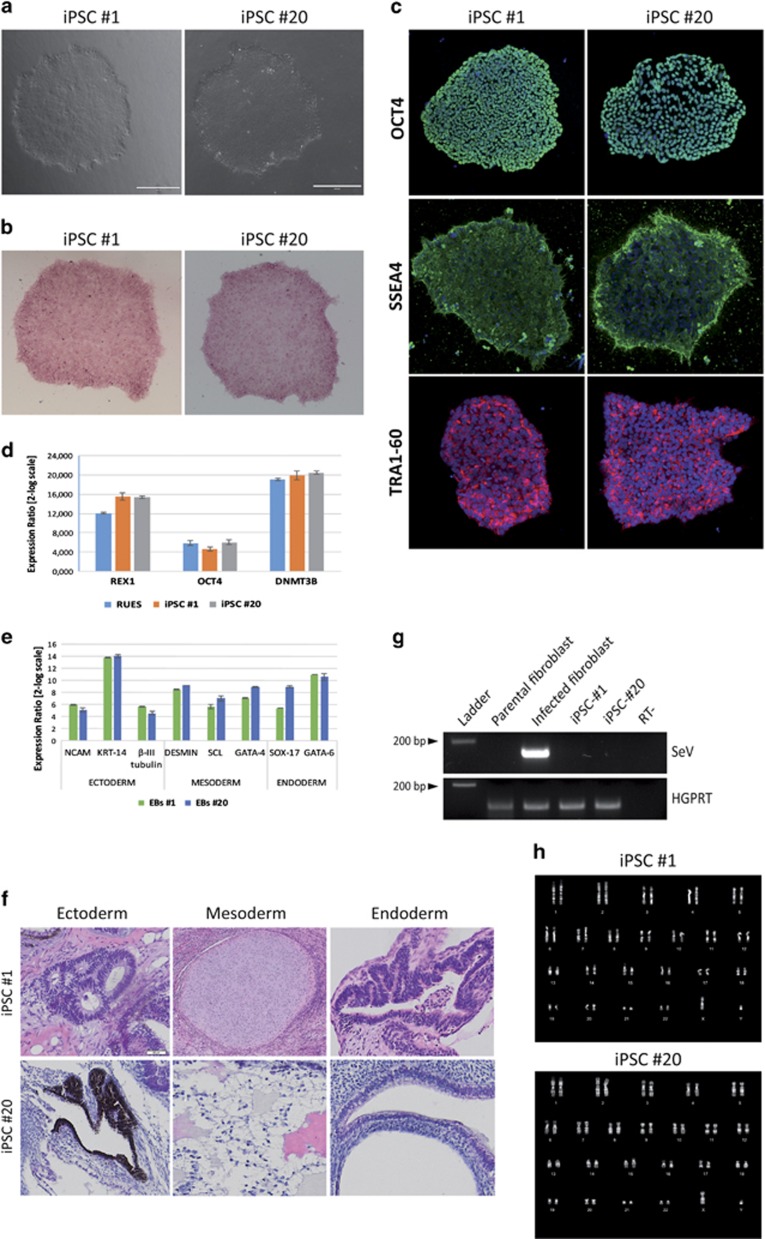
Generation of iPSCs from skin biopsy of a CPVT2 patient. (**a**) Phase-contrast images of iPSC colonies, from both clones (#1 and #20) reprogrammed from the proband B05 (HO) and subsequently used for the experiments. Scale bar: 400 *μ*m (**b**) Representative images of iPSC colonies (one each iPSC line) showing positive staining for alkaline phosphatase activity. (**c**) Immunostaining of CPVT-iPSC lines (clones #1 and #20) showing expression of stemness-specific markers OCT-4 (top), SSEA-4 (middle) and TRA1-60 (bottom). (**d**) Semiquantitative real-time PCR showing upregulation of specific markers of pluripotency (Rex-1, DNMT3B and Oct4) in two CPVT-iPSC clones (#1 and #20). The data are presented relative to parental fibroblasts and were normalized to *HGPRT* expression; RUES2 embryonic stem cell line has been used as positive control reference. Values are mean ±S.E. Diagram shows results from one representative experiment (out of three). (**e**–**f**) Evaluation of the developmental competence of CPVT-iPSC lines by embryoid bodies aggregation (**e**) and teratoma formation assay (**f**). Panel E show the results of semiquantitative real-time PCR of EBs from two CPVT-iPSC lines (#1 and #20) at d30 of differentiation and indicate upregulation of expression of markers of the three germ layers in EBs obtained from both lines. The data are relative to undifferentiated iPSC and were normalized to *HGPRT* and *18S* housekeeping genes expression. Values are mean ±S.E. Diagram shows results of one representative experiment (out of three). (**f**) Hematoxylin-eosin staining of teratomas formed by CPVT-iPSC lines injection into immunocompromised mice, showing presence of tissues that derive for all the three germ layers: neural rosettes and retinal epithelium are indicative of ectoderm formation, cartilage and adipose tissue are from mesoderm, and gut and respiratory epithelium indicate presence of endodermal differentiation. (**g**) RT-PCR against the *SeV* genome indicating loss-of-expression of the *SeV* exogenous genes in both the iPSC clones (#1 and #20) selected for the study. Parental fibroblasts and those infected with SeV genes for reprogramming have been respectively used as negative and positive controls. Detection of *HGPRT* gene expression has been used as loading control. (**h**) Representative image of the analysis of the karyotype of the iPSC lines generated from the proband, showing the reprogramming did not induce any major chromosomal abnormality

iPSC lines were generated from skin fibroblasts of both the proband who carries the mutation in homozigosity (HO) and from the healthy father (HE-heterozigous) carrier of a single copy of the same mutation, using a Sendai virus (SeV)-based system expressing the four ‘Yamanaka' factors (OCT-4, Sox2, Klf4 and c-MYC) (CytoTune 2.0 iPSC reprogramming kit from Thermo Scientific, Waltham, MA, USA). The obtained iPSC clones have been selected based on their morphological similarity to embryonic stem cells (Figure 2a; Supplementary Figure 1A) and further validated to prove their actual pluripotency. In details, we demonstrated that reprogrammed iPSC clones possess alkaline phosphatase activity (Figure 2b; Supplementary Figure 1B) and express surface markers (SSEA-4, TRA1-60) and transcription factors (OCT-4, Rex1 and Dnmt3B) that are typical of a pluripotent cell (Figures 2c and d; Supplementary Figure 1C–D). We also showed the ability of these cell lines to differentiate into derivatives of the three germ layers, both *in vitro* through embryoid bodies aggregation (Figure 2e; Supplementary Figure 1E) and *in vivo* by teratoma formation assay (Figure 2f; Supplementary Figure 1F), proving the generated models possess a complete developmental potential and therefore are fully pluripotent. Direct sequencing analysis also confirmed that the generated models were genetically matched to the donors and carried the *CASQ2* mutation (Supplementary Figure 2). We also verified that the generated iPSC lines were free of the exogenous SeV genes used for the reprogramming (Figure 2g; Supplementary Figure 1G) and maintained a normal karyotype (Figure 2h; Supplementary Figure 1H).

With the aim to develop the cardiac model of CPVT2, we next generated CMs through direct differentiation of iPSCs from HO (Supplementary Figure 3A); as a control, CMs have been also obtained from HE-iPSC lines and from WT iPSC lines we previously generated from an unrelated healthy subject.^9^

In a first instance, we verified that CMs generated from iPSCs expressed cardiac-specific proteins by checking the presence of *α*-sarcomeric actinin (Supplementary Figure 3B), as well as calsequestrin-2 (Supplementary Figure 3C) – which is normally induced at late stages during differentiation of pluripotent stem cells,^10, 18^ indicating that this model could be used as a surrogate of human CMs.

### Electrophysiological studies indicated CMs derived from CPVT2-iPSC lines recapitulate the phenotypic features typical of the disease

**Figure 3:**
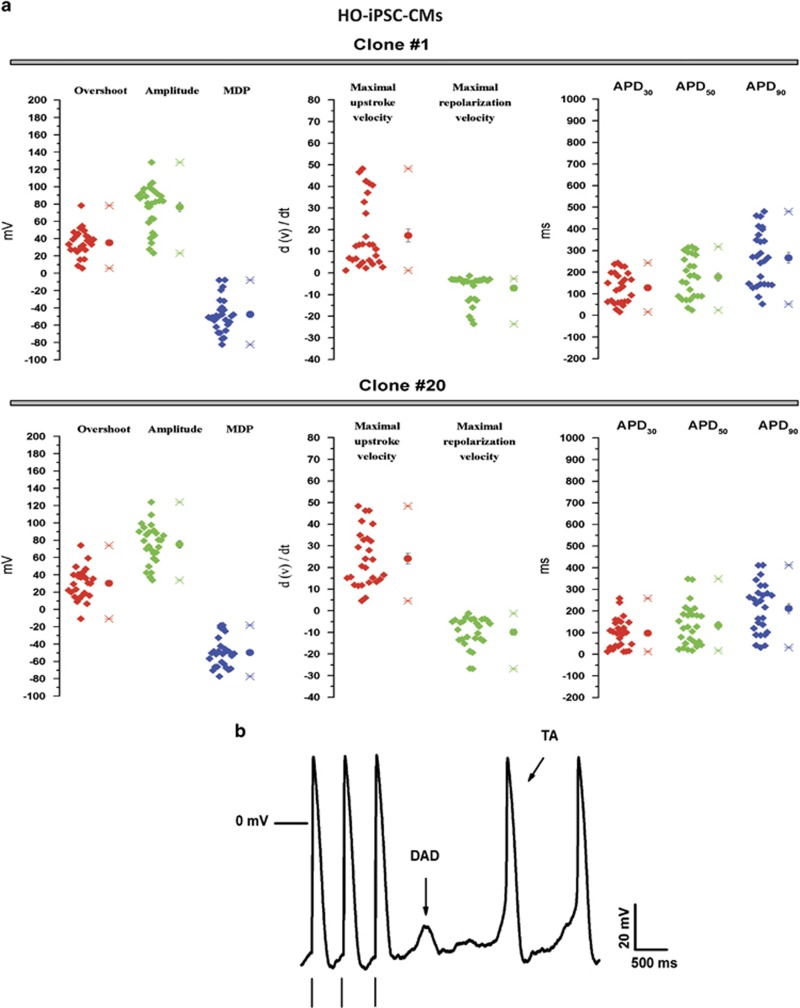
Cardiomyocytes differentiated from CPVT-iPSCs recapitulate functional defects as of CPVT2. (**a**) The main AP features measured in the HO-CMs differentiated from 2 distinct iPSC clones (#1 and #20): overshoot, amplitude, maximal diastolic potential (MDP), maximal upstroke velocity, maximal repolarization velocity and AP duration at 30, 50 or 90% of repolarization (respectively APD_30_, APD_50_ and APD_90_). Values are mean±M.S.E, Total n=55 (clone #1, *n*=27 cells; clone #20, *n*=28 cells). (**b**) Example of evoked APs recorded in HO-CMs in the presence of a *β*-adrenergic stimulus (1 *μ*M Iso); delayed occurred in 78% of the cases (DADs, 17 out of 22), while triggered activity in 12.5% (TA, 3 out of 22). Both arrhythmic events are indicated by arrows; the vertical bars under the AP indicate the stimulus. The data are relative to CMs generated from both iPSC clones

Although iPSCs are recognized as valuable tools for reproducing diseases' traits *in vitro*,^12, 15^ the demonstration that this concept applies also to our CPVT2 model is an essential requirement for the next investigations. To this purpose, we first determined the electrophysiological properties of generated CMs using the patch clamp technique. As expected, analysis of general (i.e., non CPVT-specific) action potential properties, including overshoot, amplitude, maximal diastolic potential (MDP), maximal upstroke velocity, maximal repolarization velocity and action potential duration (APD) at 30, 50 and 90% of repolarization, did not show any difference between HO-CMs and those differentiated from WT and HE lines (Figure 3a; Supplementary Figures 4A and B). Parameters were also comparable to those previously recorded in CMs from CPVT1 iPSC lines generated by us from a patient carrying the E2311D heterozygous mutation in the cardiac ryanodine receptor gene (*RyR2*).^9^ Instead, when exposed to *β*-adrenergic stimulation by isoproterenol, HO-CMs displayed delayed afterdepolarizations (DADs) and, less frequently, triggered activity (TA) during the diastolic depolarization phase (Figure 3b) that represent hallmarks of CPVT-CMs.^9, 10, 11, 12, 19^ These phenomena were absent in both WT- and HE- CMs, used as controls (Supplementary Figures 4C,D).

We also determined whether the absence of CASQ2 was affecting induction toward specific cardiac cell populations during differentiation (i.e., pacemaker cells, ventricular or atrial cells). To this purpose, we clustered CMs obtained from iPSC differentiation into two distinct populations, the nodal-like (cells from the atrio-ventricular node) and working-like myocardial cells (i.e., cells from the atrial and ventricular chamber), based on their electrophysiological properties (maximal upstroke velocity, action potential amplitude and ratio between APD_90_ and APD_50_), as previously described for the CPVT1 model.^9^ Results from these analyses revealed no significant differences between HO-, HE- and WT-iPSCs in differentiation potential and show a proportion of nodal-like cells of ~25–30% in all conditions (Supplementary Figure 5).

Altogether, these results indicate that iPCS-derived CMs replicated key features of recessive CPVT and therefore provide an *in vitro* model of the disease suitable to experiment the gene therapy approach that we have previously reported on a different human CPVT mutant.^3, 4, 5^

### AAV9-mediated CASQ2 delivery re-establish correct expression of calsequestrin-2 in CPVT2-CMs

We then aimed to determine whether AAV9-based gene delivery that effectively prevented arrhythmias in genetically-modified mice (R33Q-CASQ2 knock-in and CASQ2 knockout)^5, 6^ is also effective in patient-derived myocytes. Our idea was to test a hypothesis that the observations obtained in the CPVT mouse also apply to a human-derived CPVT model caused by a different mutation.

**Figure 4:**
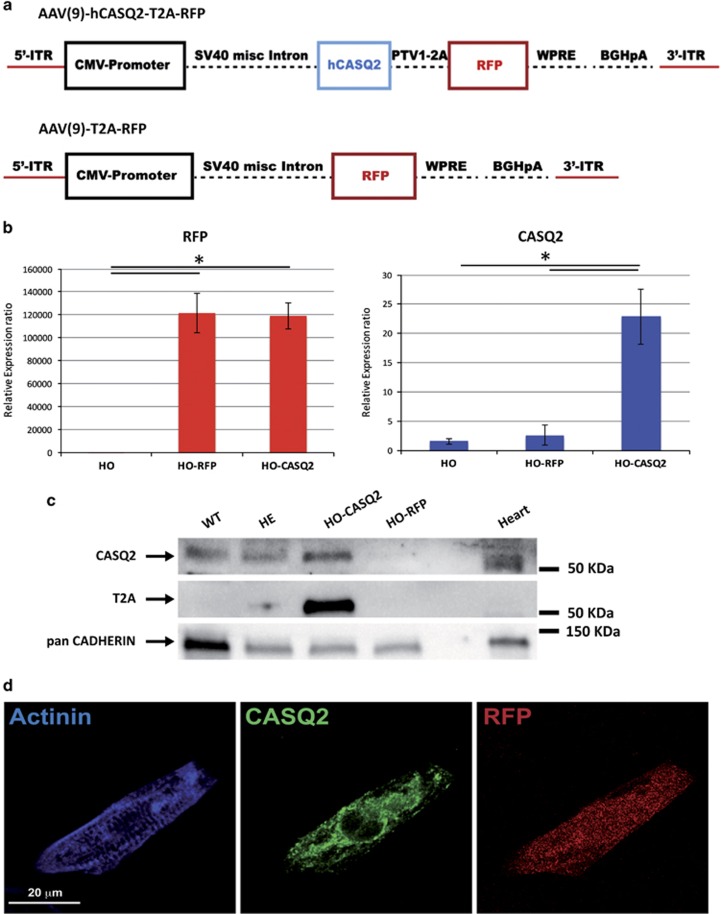
AAV9-mediated administration of WT human *CASQ2* gene re-establish physiological levels of calsequestrin-2 in HO-CMs. (**a**) Schematic representation of the adenoviral vectors (AAV9) carrying the WT human *CASQ2* gene and the RFP marker (top) or the RFP alone (bottom), used for generation of the viral particle and for the gene therapy experiments. (**b**) Semiquantitative real-time PCR showing that the infection of CPVT-CMs with AAV9-WT-hCASQ2-T2A-RFP efficiently drives overexpression of *CASQ2* gene and *RFP* in these cells (HO-CASQ2), while levels of CASQ2 in cells infected with AAV9-T2A-RFP (HO-RFP) were comparable to not infected CMs (HO). The data are relative to not infected CMs and were normalized to *HGPRT* expression. Values are mean±S.D.; *P<0.05. (**c**) western blot analysis showing re-expression of calsequestrin-2 in CPVT-CMs infected with AAV9-WT-hCASQ2 (HO-CASQ2). As expected, expression of the protein is not detectable in HO-CMs with the empty vector (HO-RFP), while the protein is present in CMs differentiated from both WT and HE iPSC lines. Protein extract from mouse heart has been used as positive control. T2A expression is instead detectable only in HO-CASQ2 CMs, indicating that the expression of the CASQ2 protein in those samples is the results of the viral-mediated gene delivery. (**d**) Immuno-staining for *α*-actinin and CASQ2 of HO-CMs infected with AAV9-WT-hCASQ2-T2A-RFP, showing positive expression of CASQ2 and positivity for RFP. Scale bar=20 *μ*m

To this aim, we took advantage of the AAV9 vector previously developed in our laboratory^5^ (AAV-hCASQ2-RFP) (Figure 4a) to re-establish expression of CASQ2 in HO-CMs.

AAV9 efficiently infected CMs as demonstrated by real-time PCR amplification of the *RFP* reporter gene (Figure 4b) and by epifluorescence (Figure 4d; Supplementary Figure 6A); infection of mutated HO-CMs induced overexpression of *CASQ2* gene (Figure 4b) and restoration of expression of the protein (Figures 4c and d), as in CMs differentiated from control iPSCs.

### Viral-mediated administration of WT human *CASQ2* gene rescues the functional defects of CPVT2-CMs

**Figure 5:**
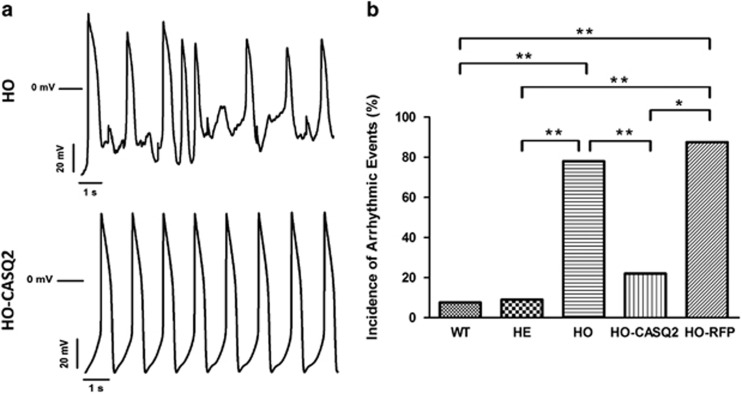
AAV9-WT-hCASQ2 infection exerts an antiarrhythmic effect on CPVT2-iPSC-derived CMs. (**a**) Representative traces of spontaneous action potentials recorded in HO-CMs prior (HO, top) and after (HO-CASQ2, bottom) administration of AAV9-WT-hCASQ2 in the presence of a *β*-adrenergic stimulus (1 *μ*M Iso). (**b**) Quantification of the incidence of Iso-induced DADs and TA, named as arrhythmic events (AE), in WT, HO, HO-CASQ2 (HO-CMs infected with the AAV9-hCASQ2-T2A-RFP vector), HO-RFP (HO-CMs infected with the empty vector) and HE CMs. (AE_WT_: 8%, 1 of 13 cells, AE_HE_: 9%, 2 of 22 cells; AE_HO_: 78%, 17 of 22 cells; AE_HO-CASQ2_: 22%, 2 of 9 cells; AE_HO-RFP_: 87.5%, 7 of 8 cells; *P<0.05, **P<0.01). The data have been generated on CMs differentiated from two independent clones from each subject

The functional properties of the CMs after administration of *CASQ2* gene therapy were then tested by action potentials and calcium dynamics assessment through patch-clamp and imaging-based techniques. AAV9-mediated expression of WT hCASQ2 was able to prevent the development of isoproterenol-induced DADs and TA in CPVT2 HO-CMs (from 78%, 17/22 in HO cells to 22%, 2/9 in HO-CASQ2, *P*<0.01 HO-CASQ2 *versus* HO) (Figure 5a); thus, the incidence of these arrhythmogenic events was reverted to levels indistinguishable to WT- and HE-CMs used as controls (Figure 5b); on the contrary, mutant HO-CMs infected with the empty AAV9-vector (HO-RFP) did not show detectable reduction of DADs and TA (Figure 5b; Supplementary Figure 6B; DADs + TA in HO-RFP: 87.5%, 7/8 cells, ns HO-RFP *versus* HO).

Spontaneous intracellular Ca^2+^ transients and Ca^2+^ sparks were also assessed after *hCASQ2* gene delivery. We measured these properties before and after adrenergic activation with 1 *μ*M isoproterenol. A summary of the measurements for all parameters and conditions is provided in the Supplementary Figure 7.

**Figure 6:**
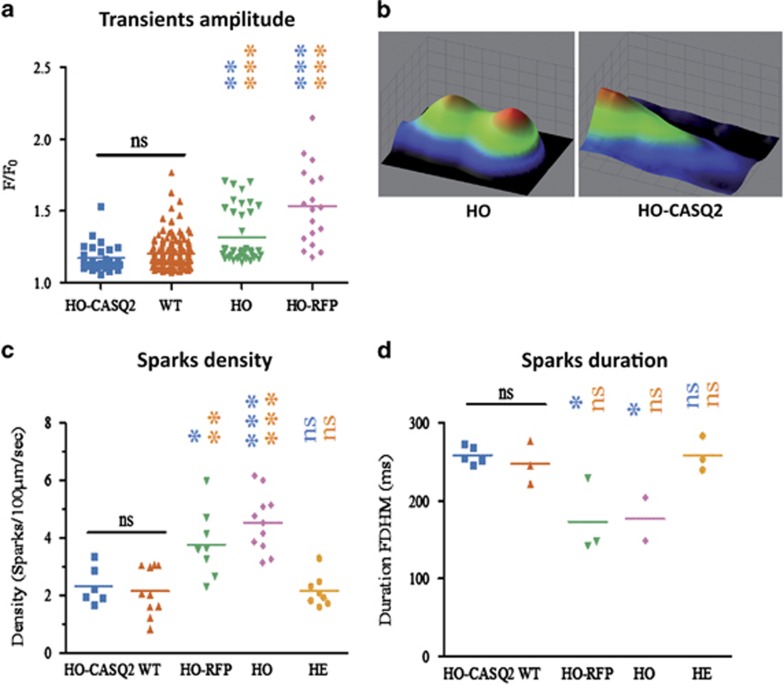
AAV9-WT-hCASQ2 infection normalizes intracellular calcium dynamics in HO-CMs. (**a**) Measurement of transient fluorescence amplitude show significantly higher transients in HO-CMs than HO-CASQ2. (**b**) Representative images of Ca^2+^ sparks represented as an X-Time 3D profile. False colors change with fluorescence intensity. (**c**, **d**) Diagrams showing measurements of calcium sparks density and durations. Calcium sparks have higher density and lower duration in HO-CMs, while CASQ2 overexpression re-establishes the phenotype to a behavior compatible with parameters' values measured in the WT cells and HE-CMs. *P*-values are color coded with the respective sample (*P<0.05; **P<0.01; ***P<0.001). The data are the mean of values registered on CMs derived from two independent iPSC lines in each condition

These experiments revealed an amelioration of Ca^2+^ transients amplitude (F/F_0_ – ratio between peak and baseline) in HO-CMs upon infection with AAV-9-hCASQ2-RFP (HO-CASQ2) (Figure 6a; Supplementary Figure 7A), which reverted to a phenotype akin to that recorded in WT-CMs (representative profiles are shown in the Supplementary Figure 7A). Average peak amplitude of Ca^2+^ transients (F/F0) in response to isoproterenol was found higher in the diseased HO-CMs compared with controls, a result similar to that shown in iPSC-CMs carrying the F2483I mutation in RyR2.^20^

Intracellular Ca^2+^ sparks density and duration (FDHM: full duration at half maximum) also significantly recovered in HO-CMs after hCASQ2 administration (Figures 6b–d); other parameters, such as Ca^2+^ sparks amplitude and size (FWHM : full width at half maximum) (see Supplementary Figure 7) were not significantly affected in mutated HO-CMs, a consequence most probably linked to the developmental heterogeneity of different iPSC lines and to the immaturity of CMs derived from pluripotent cells.^14, 18^

## Discussion

Here we demonstrate the therapeutic efficacy of *CASQ2* gene therapy in human cardiac myocytes derived from a CPVT patient presenting with a severe clinical phenotype and a radical mutation (frameshift leading to premature truncation). We do also provide the proof of concept that iPSC-CM is a robust model that allows to explore the efficacy of gene therapy on different mutations within a target gene. This is an important concept that overcomes a major limitation of preclinical studies on gene therapy that are usually performed in the few available knockin mice models. Gene replacement is ideal in homozygous conditions in which the protein is absent or non-functioning, such as described here. Our study thus provides a further rational basis for clinical gene therapy studies on CPVT2 and opens to the systematic assessment of this therapeutic strategy to multiple *CASQ2* mutations.

### Clinical implications and clinical needs

CPVT patients currently receive lifelong therapy with *β*-blockers; however, this therapy has been demonstrated to be only partially effective in preventing the development fatal arrhythmias. As much as 25–30% of patients indeed experience recurrent cardiac arrests or die suddenly while on *β*-blocker therapy over an observation time of 5 years.^21, 22^ An additional subgroup of subjects representing 5–10% of the population does not tolerate the required dose of *β*-blockers and therefore is only partially protected. For all these patients either addition of flecainide to *β*-blockers or implantation of ICDs are the recommended treatment.^23^ Although ICDs can prevent deaths, they cannot prevent the onset of arrhythmias, and their use is accompanied by adverse events, such as infections, lead fractures and inappropriate discharges. Overall, ICDs significantly affect the quality of life, especially in young children and teenagers.^24^

On the basis of strong experimental evidence, gene therapy is emerging as an attractive strategy to treat CPVT2;^4^ preclinical studies have shown that delivery of *CASQ2* gene using AAV9 vectors is sufficient to restore the normal function of calsequestrin-2 in the heart, and to prevent the onset of the disease in CPVT2 murine models.^5, 6^ Whether this effect is replicated in humans and whether it is extendable to other *CASQ2* mutations remains uncertain.

In order to answer these questions, we therefore developed a human model of CPVT2 derived from a patient carrying the G112+5X mutation in the *CASQ2* gene through reprogramming to iPSCs, a technology that has served as a tool model for evaluating potential therapies for various cardiovascular diseases, including CPVT.^9, 10, 25, 26^ Similar to mouse models, AAV9-based administration of the human *CASQ2* gene to CPVT2-CMs was sufficient to normalize the functional defect detected in those cells, demonstrating the therapeutic value of viral-based therapy of CPVT also in a human setting. These results, together with recent clinical studies in support of the safety of AAV for cardiac delivery,^27, 28, 29^ may accelerate the route toward the translational application of iPSC-based experimental models for customized preventive tests, efforts which may eventually lead to the application of gene therapy strategies in the clinical setting for patients with CPVT or other cardiovascular recessive disorders.

## Materials and Methods

### iPSC generation and differentiation into CMs

iPSCs have been generated from patients' skin fibroblasts using the CytotuneiPS-2.0 Sendai Reprogramming kit (Thermo Scientific). Reprogrammed clones were selected based on their morphology and subjected to complete validation, that included analysis expression of pluripotency markers, assessment of developmental potential *in vitro* and *in vivo,* and karyotype analysis, as described.^9^ Loss-of-expression of exogenous Sendai viral genes has been verified by RT-PCR using the following primers that specifically amplify the SeV genome (SeV_Forward primer: GGATCACTAGGTGATATCGAGC; SeV_Reverse primer: ACCAGACAAGAGTTTAAGAGATATGTATC; product size: 189 bp).

All the experiments have been conducted on two fully characterized iPSC lines from each individual.

Cardiac differentiation has been achieved using a chemically-defined serum free protocol, based on activation (CHIR99021) and inhibition (IWR1) of the Wnt pathway in RPMI-B27 medium, as previously described.^30, 31^ CMs were used for experiments in a stage of differentiation comprised between d25 and d30 (25–30 days after spontaneous contracting activity started), a differentiation stage that we showed to be sufficient to induce expression of calsequestrin-2 (as shown in the Supplementary Figure 3C). High differentiation efficiency of each iPSC line has been also verified by flow cytometry using *α*-sarcomeric actinin to specifically detect cardiac cells (Supplementary Figure 3B).

### Flow cytometry analysis

Differentiated iPSC-derived CMs were harvested and dissociated in single cell as previously described.^9^ Intracellular *α*-sarcomeric actinin staining was performed after fixation in paraformaldehyde 1% and cell permeabilization, using the appropriate saturating concentration of the unconjugated antihuman *α*-sarcomeric actinin antibody (mouse monoclonal, 1 : 400 from Abcam, Cambridge, UK). Detection was carried out using a goat anti-mouse Alexa-647-conjugated antibody (1 : 500 from Molecular Probes, Thermo Scientific). Dead cells were excluded from the analysis using LIVE/DEAD fixable aqua stain kit (Molecular Probes, Thermo Scientific). Analysis of stained cells was performed on FACS LSRFortessa flow cytometer (BD Bioscience, San Jose, CA, USA). DIVA software (BD Pharmingen, San Diego, CA, USA) was used for the data acquisition and analysis.

### Viral construct

The complete cDNA of the human cardiac *CASQ2* was cloned into a AAV9 vector in frame with the red fluorescent protein (*RFP*) gene (AAV9-hCASQ2-RFP) through a highly efficient self-cleaving 2A peptide derived from porcine Teschovirus-1, as previously described.^6, 32^

T2A sequence: GGAAGCGGAGCTACTAACTTCACGCTGCTGAAGCAGGCTGGAGACGTGGAGGAGAACCCTGGACCT.

As a negative control, an empty vector carrying the only RFP has been also generated.

Cells have been infected twice at MOI=2 × 10^5^ as previously described:^33^ two rounds of infection (6 h and overnight) have been performed on differentiated CMs immediately after beating started.

### Gene expression studies

Total RNA was extracted using Trizol Reagent (Thermo Scientific) and cDNA synthetized using the SuperScriptVilo cDNA synthesis kit (Thermo Scientific). Real-time PCR was conducted using SYBR Green chemistry (SYBR Select from Thermo Scientific) as previously described.^9^

Protein expression analysis was performed by western Blot using the following antibodies: anti-CASQ2 (ABR, Golden, CO, USA and Sigma-Aldrich, St. Louis, MO, USA), anti-T2A (Millipore, Billerica, MA, USA), anti-pan Cadherin (Sigma-Aldrich) as described.^6^

### Immuofluorescence analysis

Labeling of iPSC-derived CMs was performed after fixation (paraformaldehyde 4%) and permeabilization (Triton 0.1%) using anti-CASQ2 (ABR) and anti- *α*-actinin (Sigma-Aldrich) antibodies.^9^ Alexa-Fluor 488- and 555- conjugated secondary antibodies were used for specific detection. Nuclei were stained with 40,6-diamidino-2-phenylindole (DAPI) and image acquired on Leica TCS-SP2 digital scanning confocal microscope (Leica Microsystems, Wetzlar, Germany).

### Electrophysiological studies

iPSC-CMs were enzymatically dissociated and seeded at low confluence on Permanox slides (Lab-Tek II from Sigma-Aldrich) previously coated with fibronectin (5 *μ*g/cm^2^, from Sigma-Aldrich). Action potentials (APs) were recorded using the patch clamp technique in the whole-cell configuration with a MultiClamp 700B (Axon Instruments, Sunnyvale, CA, USA). The experiments were performed at 37°C under continuous perfusion of extracellular solution containing (in mM) 140 NaCl, 4 KCl, 2 CaCl_2_, 1 MgCl_2_, 10 HEPES, and 5 glucose (pH adjusted to 7.40 with NaOH). Patch clamp pipettes, formed from borosilicate glass with a P-97 horizontal puller (Sutter Instruments, Novato, CA, USA), and had a resistance of 2–3 MΩ when filled with an intracellular solution containing (in mM) 20 KCl, 120 K-aspartate, 1 MgCl_2_, 4 Na_2_-ATP, 0.1 GTP, 10 glucose, and 10 HEPES (pH adjusted to 7, 20 with KOH). Some experiments were carried out with intracellular electrophysiology recordings. In this case, spontaneously beating clusters were impaled using sharp glass microelectrodes with resistances ⩾10 MΩ. Electrode capacitance was nulled and the recordings were made using the previously described MultiClamp 700B amplifier in gap-free mode.

Solutions containing isoproterenol (Iso, 1 *μ*M) were prepared fresh before the experiments and applied using a gravitational flow system for 2–3 min prior to the data collection. All signals were acquired at 10 KHz, digitized (Digidata 1332A, Axon Instruments), and analysed with pCLAMP 9.2 software (Axon Instruments). We defined delayed DADs as low-amplitude depolarizations following completion of repolarization, and have an amplitude ⩾5% of the preceding AP. Triggered activity (TA) was defined as an AP developing from a DAD rather than from an external stimulus.

### Calcium imaging measurements

Ca^2+^ transients and Ca^2+^ sparks were acquired using a TrimScope II upright two-photon microscope (LaVision Biotec, Germany) with a 60 × LUMPFL NA 1.1 (Olympus, Japan) water immersion objective, as previously described.^34^ iPSC-CMs were seeded onto 35 mm dishes and the next day loaded with 5 *μ*M Fluo-4 AM dye (Thermo Scientific) for 20 min. During acquisition, cells were kept under physiological condition with a perfusion pump in a buffer heated at 37°C. Ti : Sa infrared laser was tuned at 810 nm, and fluorescence light was separated from excitation light with a SP700 filter, then collected with a 525/50 emission filter and a non-descanned GaAsP detector (H7422-40, Hamamatsu Photonics, Japan) . After a first 512-pixel-wide XY image for each cell, we drew line ROIs and acquired 5000 XT line scans per ROI with a 0.375 um pixel size and 300 Hz time resolution, during which some calcium transients and some sparks occurred. Calcium transients were processed with Fiji/ImageJ (http://fiji.sc/) to generate table intensity data over time, then R statistical software (R Foundation for Statistical Computing, Vienna, Austria) was used to isolate single transients and measure normalized transient amplitudes. Minimum five transients per condition were analyzed. Calcium sparks were analyzed with SparkMaster ImageJ plugin (https://sites.google.com/site/sparkmasterhome/). Statistical analysis of both transient and sparks measurements was conducted in Graphpad Prism 4.03 (Graphpad Software Inc, La Jolla, CA, USA), One-Way ANOVA and Tukey multiple post-test (*P<0.05, **P<0.01, ***P<0.001).

200–580 sparks were analyzed over a minimum of 25 cells per condition.

### Statistical analysis

The data are represented as mean±M.S.E (or mean±S.D. where indicated). The significance of differences between the two groups was evaluated with unpaired Student's *t*-test. *P*<0.05 was considered statistically significant. (*) indicates *P*<0.05, (**) refers to *P*<0.01, while ns is for not significant.

### Human subjects

Approval for the use of human samples in agreement with the protocols described here has been obtained by Fondazione Maugeri Review Board (ID. 921CEC 06/14/2013).

Skin biopsies have been carried out using routine surgical techniques, without excision of excess tissue. Tissue specimens were collected in sterile saline solution and processed for isolation of fibroblast within the following 24 h.

## Abbreviations

CPVT: catecholaminergic polymorphic ventricular tachycardia type
CPVT1: catecholaminergic polymorphic ventricular tachycardia type 1 (autosomal dominant)
CPVT2: catecholaminergic polymorphic ventricular tachycardia type 2 (autosomal recessive)
hCASQ2: human Calsequestrin-2
CMs: cardiomyocytes
iPSCs: induced pluripotent stem cells
SeV: Sendai Virus
AAV9: adeno-associated viral vector serotype 9
AAV9-h*CASQ2*: AAV9 encoding the human *CASQ2* gene
SR: sarcoplasmic reticulum
ICDs: implantable cardioverter-defibrillators
RyR2: ryanodine receptor
CaMKII: Calcium–Calmodulin-dependent kinase II
RFP: red fluorescent protein
HO: homozygous
HE: heterozygous
MDP: maximal diastolic potential
APD: action potential duration
DADs: delayed afterdepolarizations
TA: triggered activity
AE: arrhythmic events
HO-RFP: HO-CMs infected with the empty AAV9-vector
HO-CASQ2: HO-CMs infected with the AAV9-hCASQ2-RFP vector
FDHM: full duration at half maximum
FWHM: full width at half maximum
F/F0: average peak amplitude of Ca^2+^ transients

## Notes

Dr Priori, Dr Napolitano and Dr Denegri own stocks of Audentes Therapeutics. Dr Lodola, Dr Morone, Dr Bongianino, Dr Nakahama, Dr Rutigliano, Dr Gosetti, Dr Rizzo, Dr Vollero, Dr Buonocore, Dr Condorelli and Dr Di Pasquale declare no conflict of interest.
