# Supplementary material for: Adeno-associated virus-mediated CASQ2 delivery rescues phenotypic alterations in a patient-specific model of recessive catecholaminergic polymorphic ventricular tachycardia
Source: Cell Death Dis. 2016 Oct 6;7(10):e2393–. doi: 10.1038/cddis.2016.304 (PMC5133973; doi:10.1038/cddis.2016.304)
Supplement: Supplementary Information [file cddis2016304x1.docx]

**SUPPLEMENTARY MATERIAL**

**Supplementary Figure Legends**

**Supplementary Figure 1. Generation of iPSC lines from heterozygous control.** A) Phase contrast images of iPSC colonies, from both clones (#1 and #6) reprogrammed from the proband’s father (B04), heterozygous carrier of the G112+5X mutation (HE). Scale bar: 400μm. B) Representative images of a staining detecting alkaline phosphatase activity in iPSC lines derived from HE. C) Immunostaining of HE-iPSC lines showing expression of specific markers of pluripotency OCT4 (top), SSEA4 (middle) and TRA1-60 (bottom). D) Semi-quantitative real-time PCR showing up-regulation of *stemness* markers (Rex-1, DNMT3B and Oct4) in two HE-iPSC clones (#1 and #6). Data are presented relative to parental fibroblasts and were normalized to HGPRT expression; RUES2 embryonic stem cell line has been used as positive control reference. Values are mean±SE. Diagram shows results from one of three independent experiments. E) Semi-quantitative real-time PCR of embryoid bodies (EBs) generated from the two HE-iPSC lines (#1 and #6) showing up-regulation of genes typical of the three germ layers. NCAM, KRT-14 and βIII-tubulin are indicative of ectodermal cells; DESMIN, SCL and GATA 4 are mesodermal markers and SOX17 and GATA6 are endodermal markers. Data are relative to undifferentiated iPSC and were normalized to HGPRT housekeeping gene expression. Values are mean±SE. A representative of three independent experiments is shown. F) Hematoxylin-eosin staining of teratomas isolated from immunocompromised mice injected with HE-iPSC lines showing their ability to generated tissues that derive for all the three germ layers: retinal epithelium and neural rosettes are indicative of ectoderm formation, cartilage and adipose tissue are from mesoderm and intestinal epithelium indicates presence of endodermal differentiation. G) RT-PCR directed against the SeV genome indicating loss of expression of the SeV exogenous genes in both the iPSC clones (#1 and #6) selected for the study. Parental fibroblasts and those infected with SeV genes for reprogramming have been respectively used as negative and positive controls. Detection of HGPRT gene expression has been used as loading control. H) Representative images of the karyotype of HE-iPSC lines, showing the cells did not carry any major chromosomal abnormality.

**Supplementary Figure 2. CASQ2 genetic analysis of HO- and HE-derived iPSC lines.** Genotyping of the generated HO- and HE-iPSC lines. The panel A shows the sequence chromatogram from a representative HO-iPSC line and confirm the line carries the same mutation of the donor, a homozygous deletion of 16 bases in position 339 of the CASQ2 gene, leading to the insertion of a stop codon. The same analysis referred to the lines derived from the father is shown in the panel B and indicate the presence of the same mutation in the heterozygous state.

**Supplementary Figure 3. Cardiac differentiation of iPSC lines.** A) Schematic representation of the differentiation protocol used to generate CPVT-CMs, based on the modulation of the Wnt pathway (rapid activation with CHIR99021, followed by a longer inhibition with IWR-1) in chemically defined, serum-free RPMI-B27 medium. Cells were analyzed 25-30 days after the appearance of contraction (usually occurring between d7 and d10 of differentiation). B) FACS analysis targeting the typical cardiac-specific structural marker α-sarcomeric actinin in CMs derived from WT- HE- and HO-iPSC lines indicating a differentiating efficiency higher than 80% for all analyzed clones. A representative density plot each line is shown. C) Western Blot analysis showing expression of calsequestrin-2 in CMs differentiated from WT and HE control iPSC lines at d25 and d30 of differentiation (25 and 30 days after beating appears). Sample from mouse heart has been used as positive control.

**Supplementary Figure 4. Electrophysiological characterization of HE- and WT-CMs.** A,B) Scatter plots with the main action potential features measured in the HE-(A) and WT-CMs (B): overshoot, amplitude, maximum diastolic potential (MDP), maximal upstroke velocity, maximal repolarization velocity and action potential duration at 30%, 50% or 90% of repolarization (APD_30_, APD_50_ and APD_90_ respectively). Values are mean±MSE. HE: n=33 cells (clone #1, n=14 cells and clone #6, n=17 cells); WT: n=29 cells (clone #1, n=13 cells and clone #2, n=16 cells). C,D) Examples of spontaneous action potentials recorded in HE- (C) and WT-CMs (D) in the presence of a β-adrenergic stimulus (1µM Iso); DADs occurred in the 9% (2/22 cells) and 8% (1/13 cells) of the cases respectively, while triggered activity was not detected in both cases (TA_HE_, 0/22 cells and TA_WT_, 0/13 cells).

**Supplementary Fig. 5: iPSC-derived CMs exhibit distinct nodal- and working-like cell populations.** Histogram plots indicating the number of nodal-like (black bars) and working-like (white bars) cells in the CMs preparations differentiated from WT- (A), HO- (B) and HE-iPSC lines (C). The classification was based on the maximal upstroke velocity (dV/dtmax ≤5 V/s for nodal-like CMs, and ≥10 V/s for working-like CMs) and on the AP morphology as described previously (9). D) Table summarizing the main AP parameters for each phenotype. Values are mean ± MSE and refer to CMs generated from two independent clones each individual. ** represents p<0.01, working- vs nodal-like.

**Supplementary Figure 6. Functional characterization of AAV9*-*RFP infection in HO-CMs.** A) Epifluorescence image of CMs infected with the RFF empty vector (pAAV9-T2A-RFP), analyzed by electrophysiology. B) Representative traces of evoked action potentials recorded in HO-CMs infected with the empty vector (in the text referred as HO-RFP). DADs occurred in the 87.5% of the cases (DADs, 7/8 cells), while triggered activity in the 37.5% (TA, 3/8 cells). Both arrhythmic events are indicated by the arrows; the vertical bars under the AP indicate the stimulus.

**Supplementary Figure 7. Complete calcium measurements.** A) Representative calcium transients’ traces recorded from line scans. CASQ2 overexpression reverts the amplitude phenotype of transients’ peaks. B, C) Graphical representation of sparks amplitude and spatial size measurements recorded after adrenergic stimulation by isoproterenol 1μM. No significant changes have been found for these parameters. D) Table summarizing measurement of calcium sparks parameters, both in basal conditions and after adrenergic stimulus. Represented data are the mean of values registered on CMs derived from two independent iPSC clones each condition.
