# Supplementary figures and images for: Adeno-associated virus-mediated CASQ2 delivery rescues phenotypic alterations in a patient-specific model of recessive catecholaminergic polymorphic ventricular tachycardia
Source: Cell Death Dis. 2016 Oct 6;7(10):e2393–. doi: 10.1038/cddis.2016.304 (PMC5133973; doi:10.1038/cddis.2016.304)

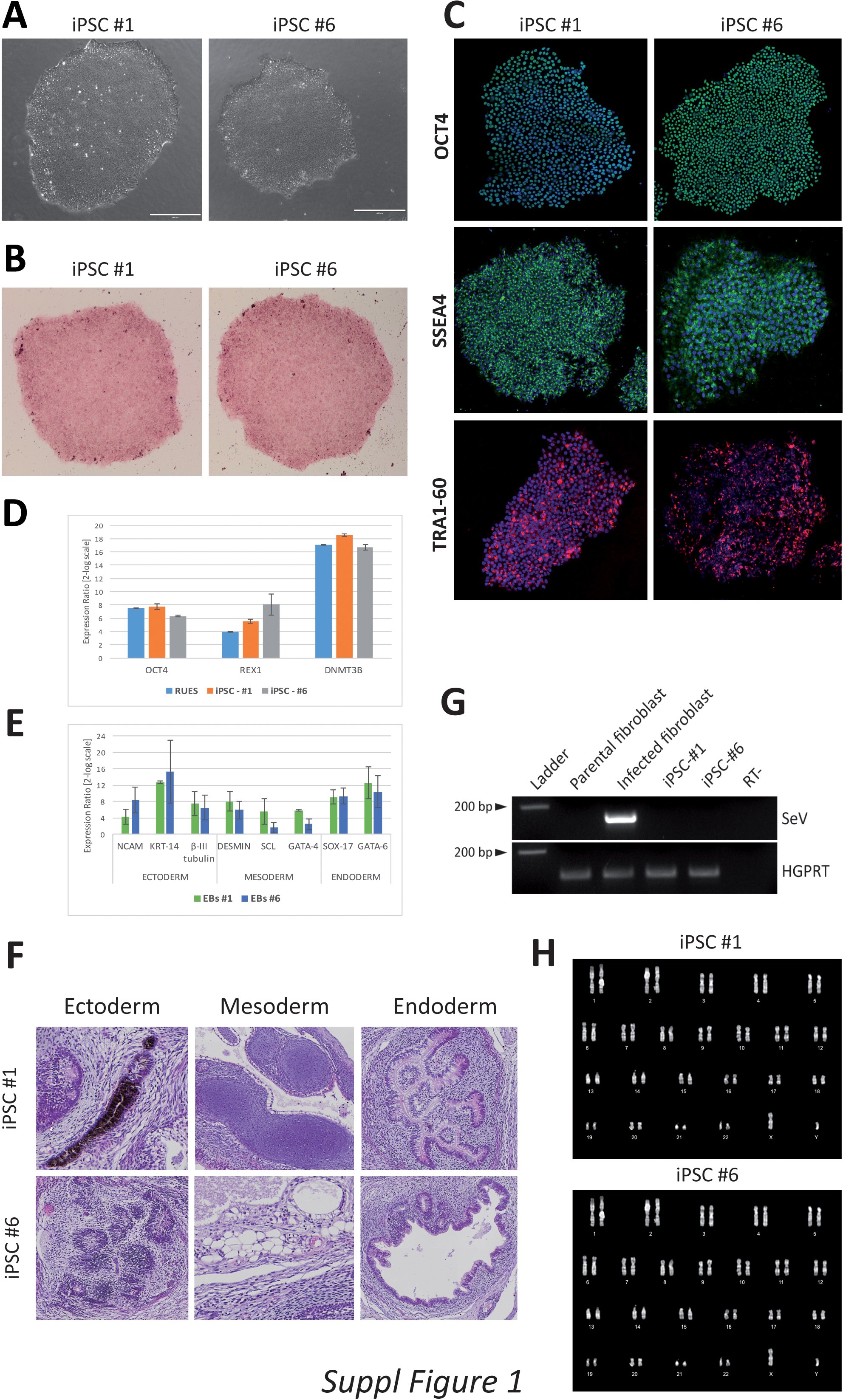

Supplement: Supplementary Figure S1 [file cddis2016304x2.tif]

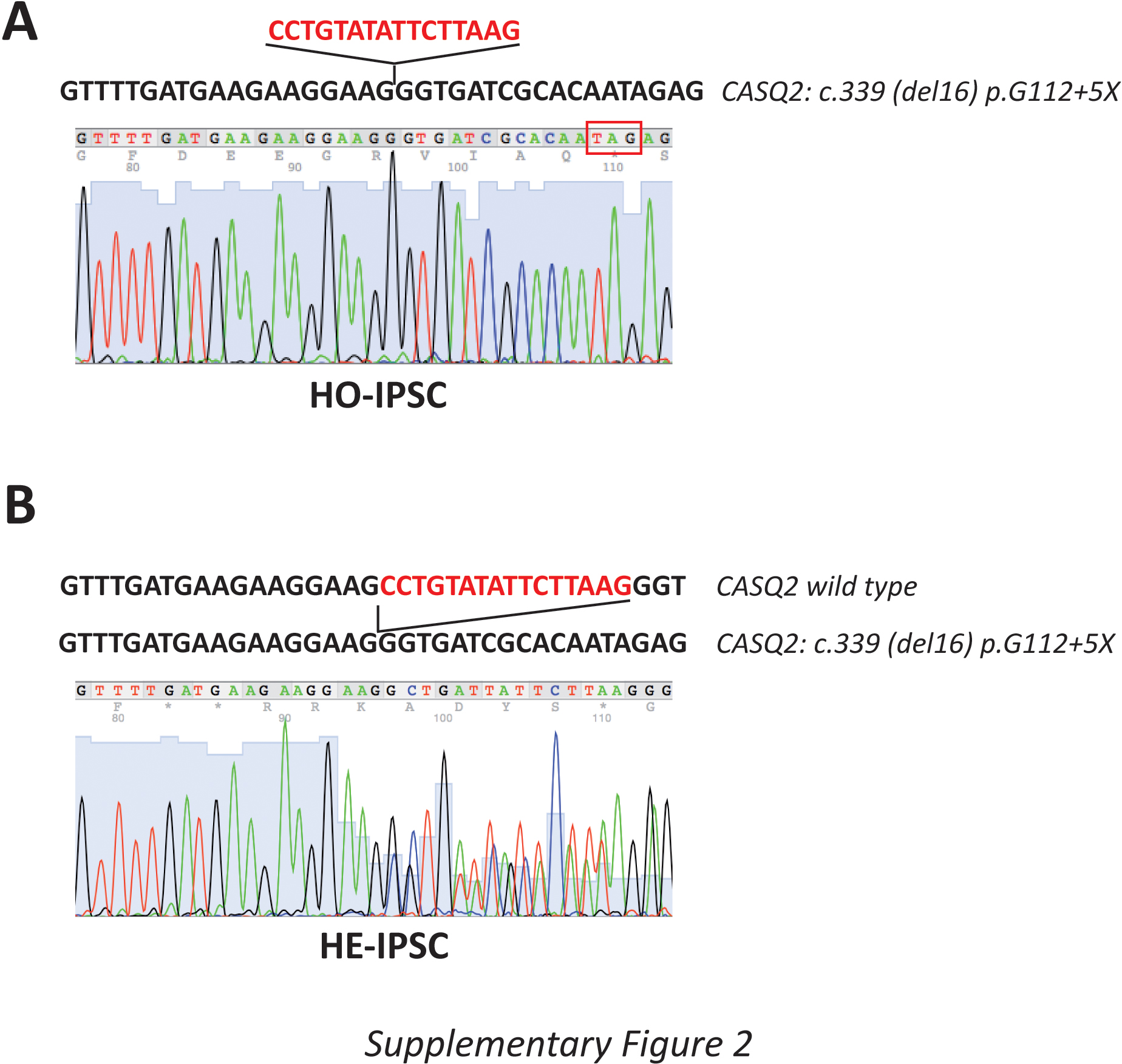

Supplement: Supplementary Figure S2 [file cddis2016304x3.tif]

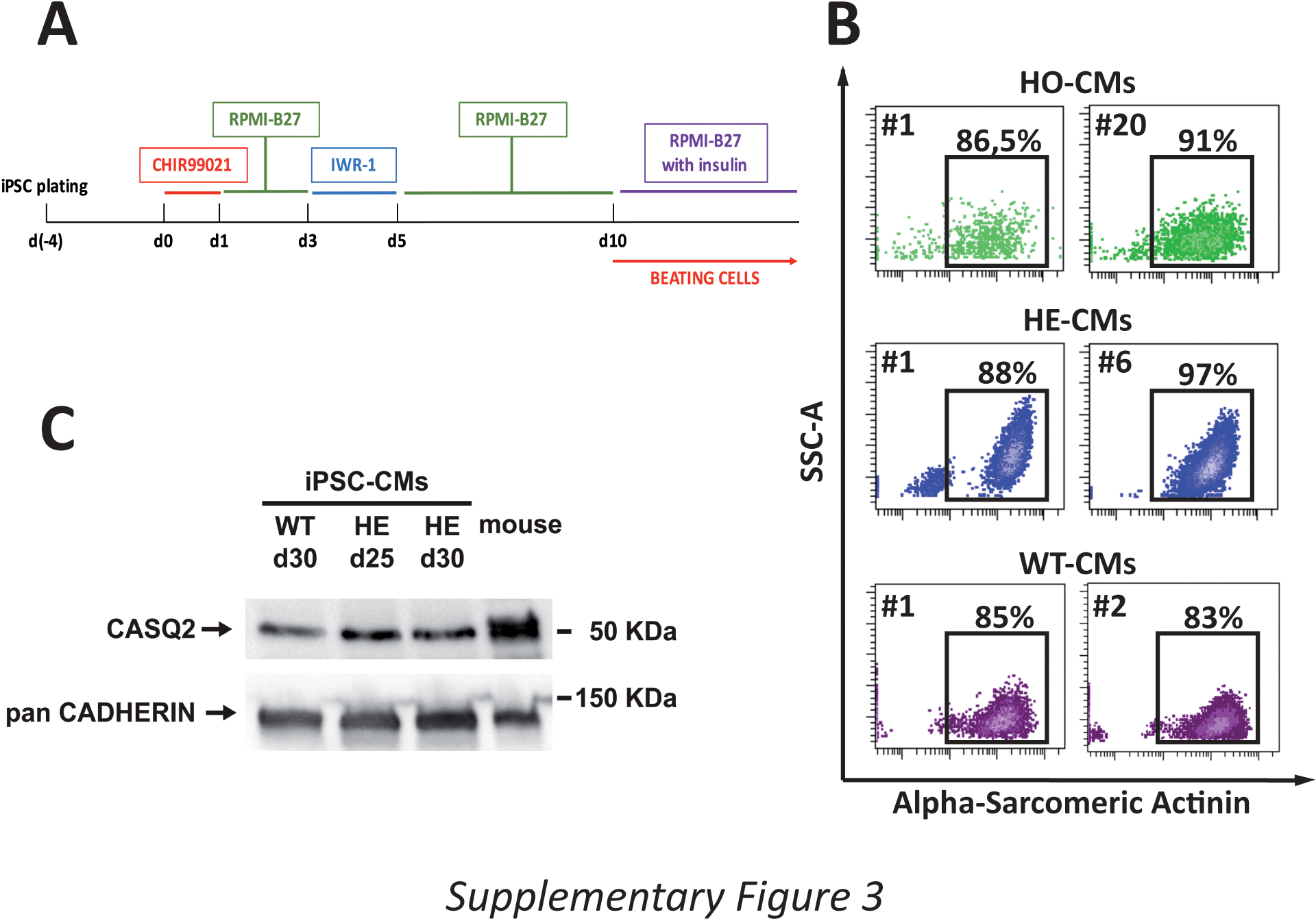

Supplement: Supplementary Figure S3 [file cddis2016304x4.tif]

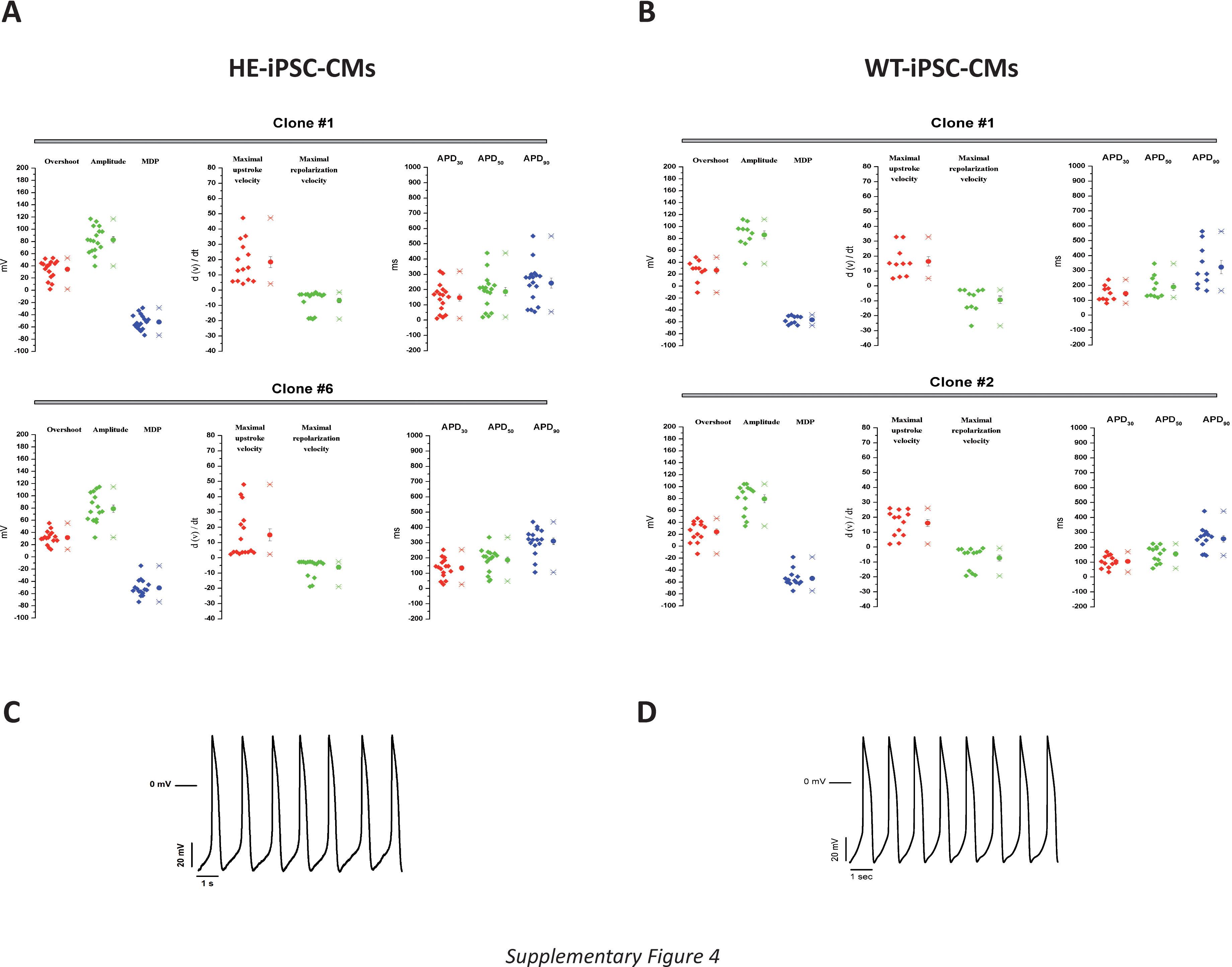

Supplement: Supplementary Figure S4 [file cddis2016304x5.tif]

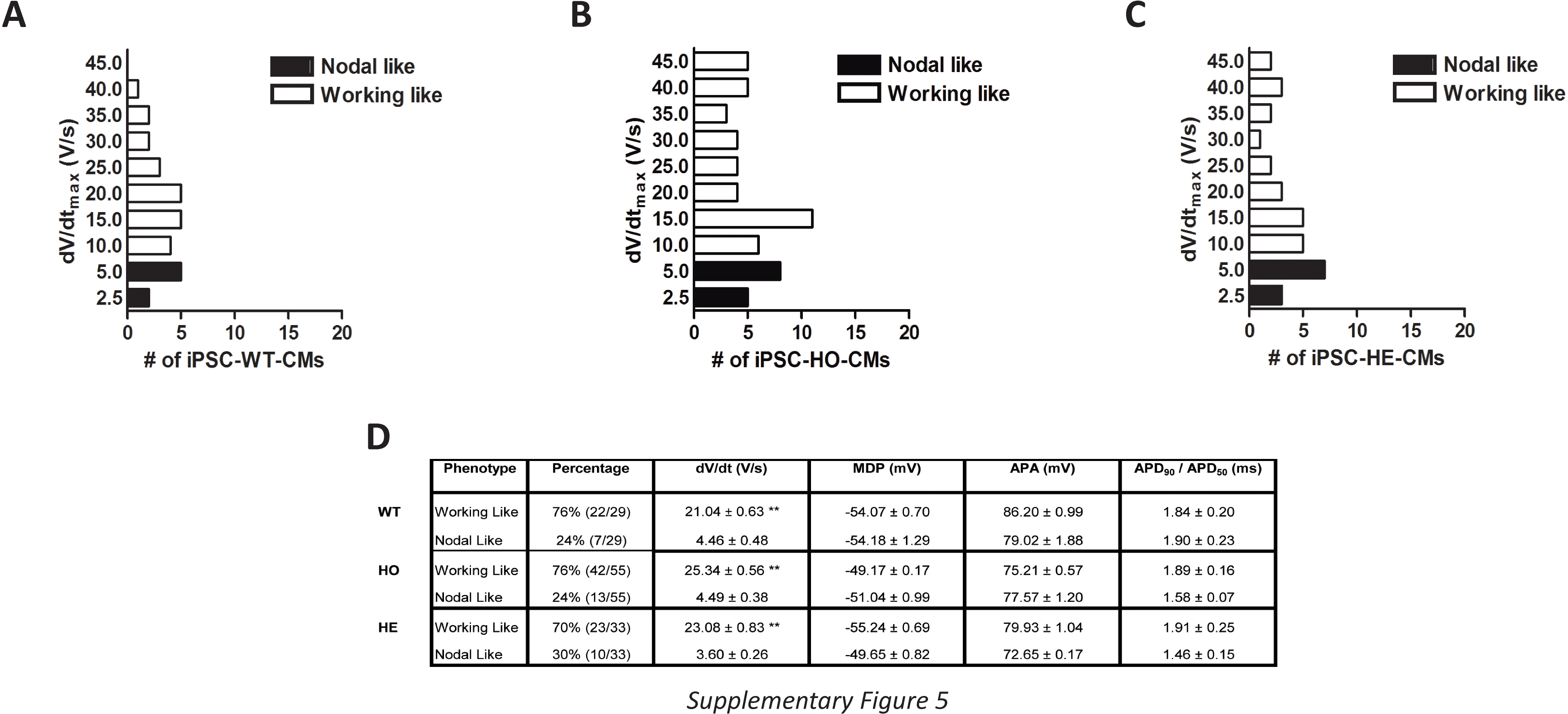

Supplement: Supplementary Figure S5 [file cddis2016304x6.tif]

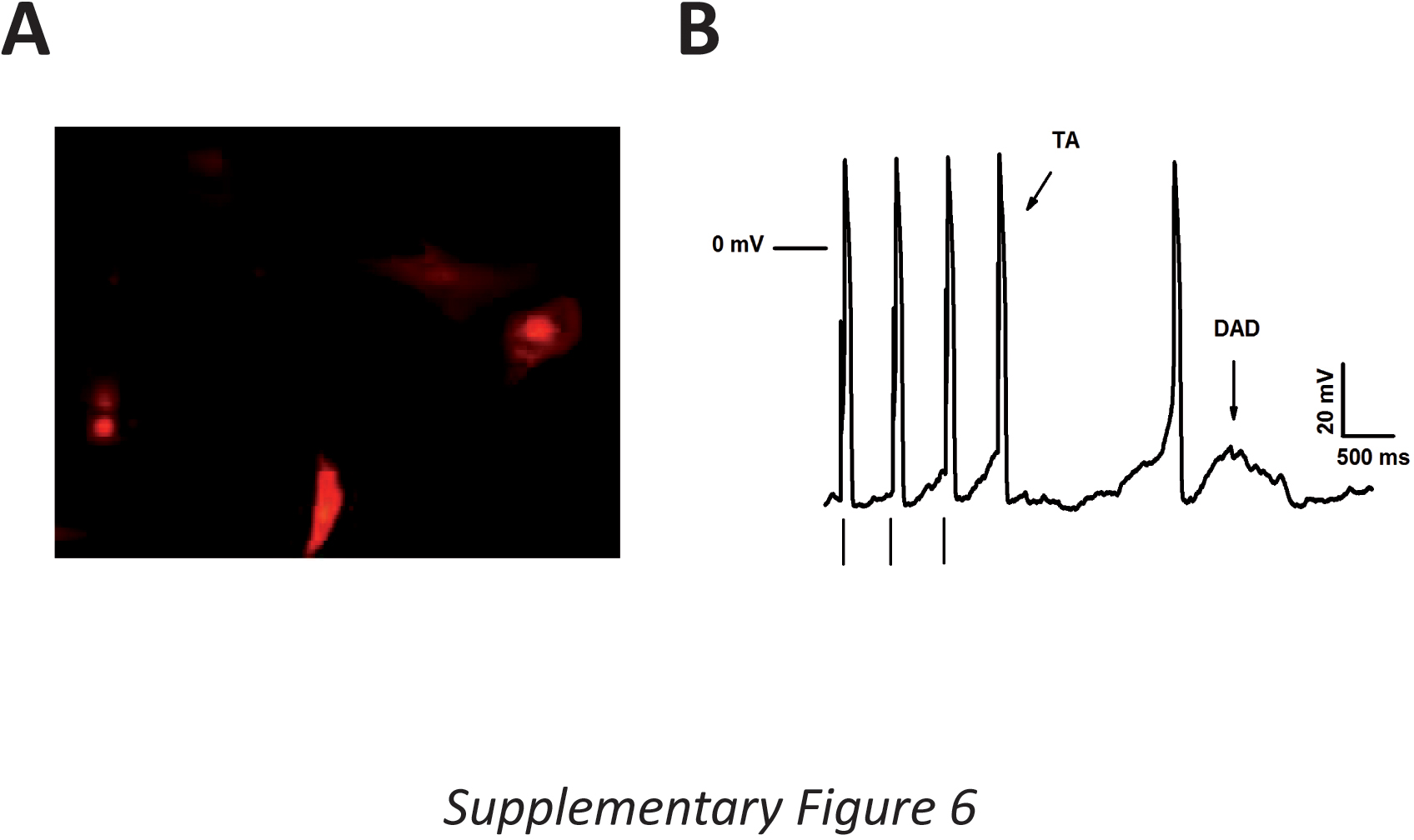

Supplement: Supplementary Figure S6 [file cddis2016304x7.tif]

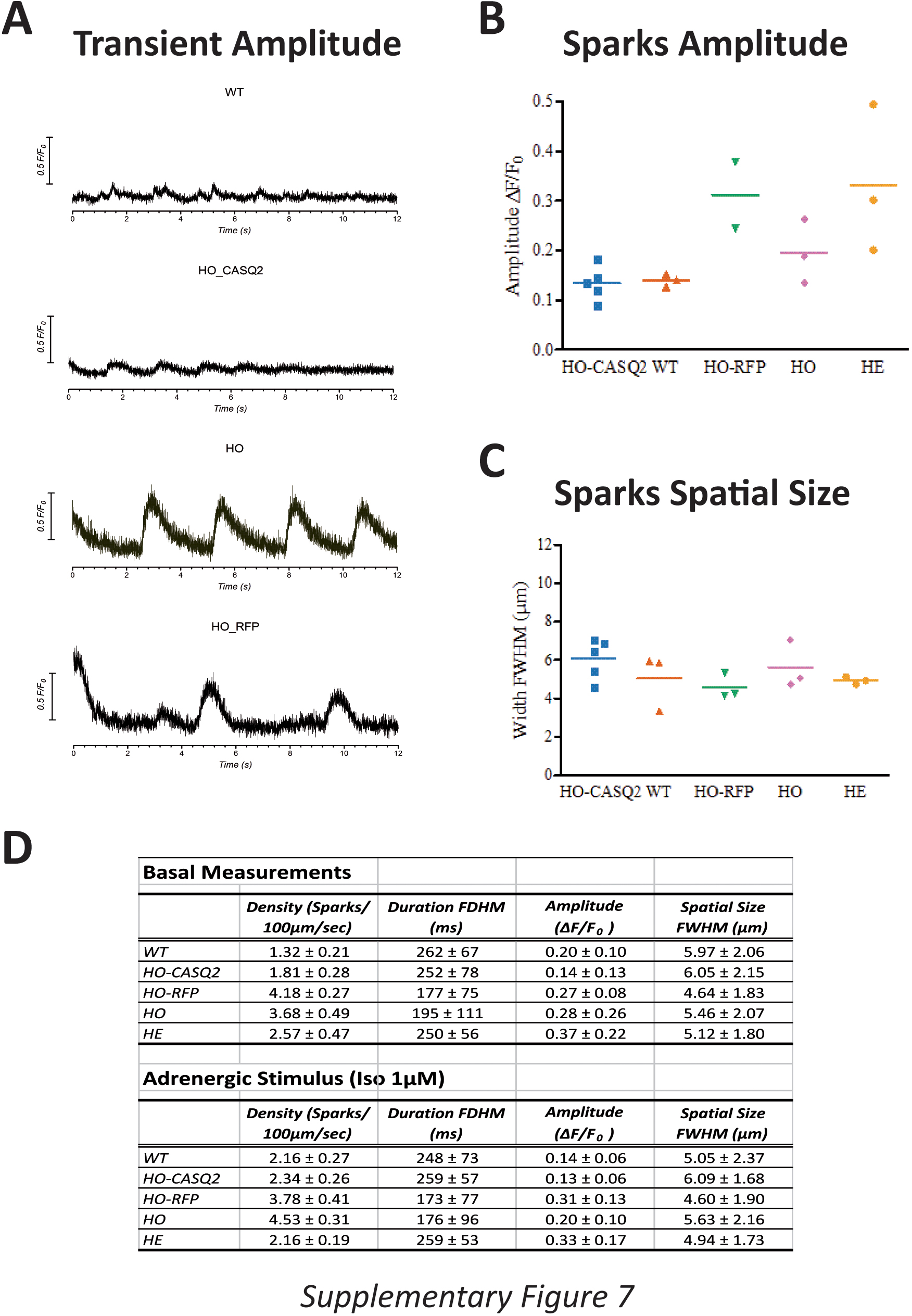

Supplement: Supplementary Figure S7 [file cddis2016304x8.tif]
